# Therapeutic Potential for Regulation of the Nuclear Factor Kappa-B Transcription Factor p65 to Prevent Cellular Senescence and Activation of Pro-Inflammatory in Mesenchymal Stem Cells

**DOI:** 10.3390/ijms22073367

**Published:** 2021-03-25

**Authors:** Rocío Mato-Basalo, Miriam Morente-López, Onno J Arntz, Fons A. J. van de Loo, Juan Fafián-Labora, María C. Arufe

**Affiliations:** 1 Grupo de Terapia Celular y Medicina Regenerativa, Departamento de Fisioterapia, Ciencias Biomédicas y Medicina, Universdidade da Coruña, 15006 A Coruña, Spain; rocio.mato.basalo@udc.es (R.M.-B.); miriam.morente.lopez@udc.es (M.M.-L.); 2 Experimental Rheumatology, Department of Rheumatology, Radboud University Medical Center, 6525 GA Nijmegen, The Netherlands; Onno.arntz@radboudumc.ni (O.J.A.); Fons.vandeLoo@radboudumc.nl (F.A.J.v.d.L.)

**Keywords:** mesenchymal stem cells, inflamm-aging, cellular senescence, SASP, sEV

## Abstract

Mesenchymal stem cells have an important potential in the treatment of age-related diseases. In the last years, small extracellular vesicles derived from these stem cells have been proposed as cell-free therapies. Cellular senescence and proinflammatory activation are involved in the loss of therapeutic capacity and in the phenomenon called inflamm-aging. The regulators of these two biological processes in mesenchymal stem cells are not well-known. In this study, we found that p65 is activated during cellular senescence and inflammatory activation in human umbilical cord-derived mesenchymal stem cell. To demonstrate the central role of p65 in these two processes, we used small-molecular inhibitors of p65, such as JSH-23, MG-132 and curcumin. We found that the inhibition of p65 prevents the cellular senescence phenotype in human umbilical cord-derived mesenchymal stem cells. Besides, p65 inhibition produced the inactivation of proinflammatory molecules as components of a senescence-associated secretory phenotype (SASP) (interleukin-6 and interleukin-8 (IL-6 and IL-8)). Additionally, we found that the inhibition of p65 prevents the transmission of paracrine senescence between mesenchymal stem cells and the proinflammatory message through small extracellular vesicles. Our work highlights the important role of p65 and its inhibition to restore the loss of functionality of small extracellular vesicles from senescent mesenchymal stem cells and their inflamm-aging signature.

## 1. Introduction

Mesenchymal stem cells (MSCs) are stromal stem cells from adult tissues, such as bone marrow, adipose tissue, the umbilical cord and others [1]. They have a high potential to be used as treatments for age-related diseases, because they are multipotent and can differentiate cell types from the mesoderm lineage [2]. The cells can easily expand in vitro, but aging affects the self-renewal capacity of MSCs [3,4]. During the aging, there are two important processes (cellular senescence and proinflammatory activation) involved in the loss of promising characteristics of MSCs in cell-based therapy [5]. Cellular senescence and proinflammatory activation (PA) produce a persistent low-grade systemic status called inflamm-aging that is associated with the degeneration of organs and tissues. The consequence of this biological process is the development of age-related diseases [6].

During the aging process, there exists an accumulation of senescent cells associated with the developing of age-related diseases [7]. Cellular senescence is a process characterized by the loss proliferation capacity and elevated lysosomal activity detected by the β-galactosidase (β-*g*al) activity [8]. Besides, senescent cells produce and deliver cytokines, chemokines, lipid mediators, metabolites and small extracellular vesicles (sEV) called senescence-associated secretory phenotypes (SASP) [9]. The conditioned medium from senescent cells can induce senescence in neighboring cells by way of the paracrines [10]. Extracellular vesicles (EV) are biological, cellular particles that are contained in bodily fluids [11]. In the last years, our group has reported that sEV are involved in the transmission of the senescence message by regulation of the inflammatory pathways (Toll-like receptor type 4 [4], mTOR [12] and interferon pathways [10]). Besides, the modification of miRNAs or proteins contained in EV can be used as antiaging therapy [12,13,14]. For example, sEV can ameliorate the mTOR pathway involved in the aging of MSCs [12] and have an important therapy in neurogenerative [15] and cardiovascular diseases [16].

The NF-kB family is integrated by five transcription factors (p50, p52, p65, c-REL and ReIB) implicated in immune and inflammatory responses [17]. The NF-kB pathway can be activated by many stimuli (cytokines, oncogenes, oxidative stress and DNA damage) [18,19]. The ReIA/p65 complex is involved in the canonical pathways NF-kB is involved in: (1) the maintained cellular senescence in mouse embryonic fibroblasts (MEFs) [20], (2) the proinflammatory activation by tumor necrosis factor α (TNF-α) in human MSCs [21] and (3) the activation of immune cells during aging [22,23]. We proposed to study the role of the p65 pathway in the cellular senescence and proinflammatory activation of MSCs and their intercellular communication by sEV using small inhibitors (JSH-23, MG-132 and curcumin).

## 2. Results

### 2.1. p65 Is Involved in the Cellular Senescence Establishment and Proinflammatory Activation in MSCs

**Figure 1:**
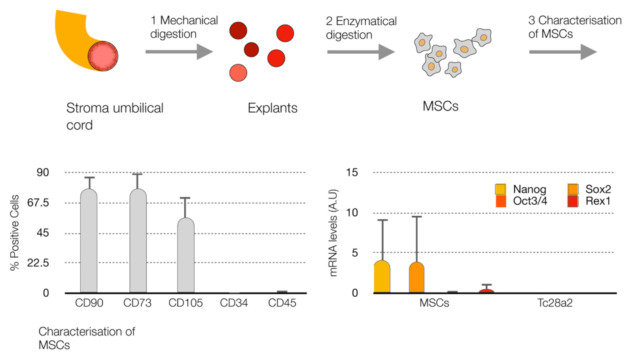
Characterization of mesenchymal stem cells (MSCs). Workflow of the isolation of MSCs from the stroma umbilical cord (**up**), histogram of the % of positive cells for the mesenchymal markers (CD90, CD73 and CD105) and hematopoietic markers (CD34 and CD45) using FACS (**bottom left**). Levels of markers for the cell undifferentiated state (*Nanog*, *Oct3/4*, *Sox2* and *Rex1*) at mRNA by qPCR-RT (**bottom right**) in MSCs and healthy chondrocytes (TC28a2).

In order to study the p65 pathway in inflamm-aging, we selected MSCs from the umbilical stroma cord (UC-MSCs) characterized by the evaluation of mesenchymal and haematopoietic markers using flow cytometry. The MSCs were positive for mesoderm markers (CD90, CD73 and CD105), and the percentage of positive cells was less than 1% for the hematopietic markers (CD34 and CD45) (Figure 1). Additionally, we evaluated the undifferentiated markers (*Nanog*, *Sox2*, *Oct3/4* and *Rex1*) at the RNA level in MSCs in comparison to healthy chondrocytes (TC28a2). The MSCs showed a high-level expression of markers for the cell undifferentiated state compared to healthy chondrocytes (Figure 1).

**Figure 2:**
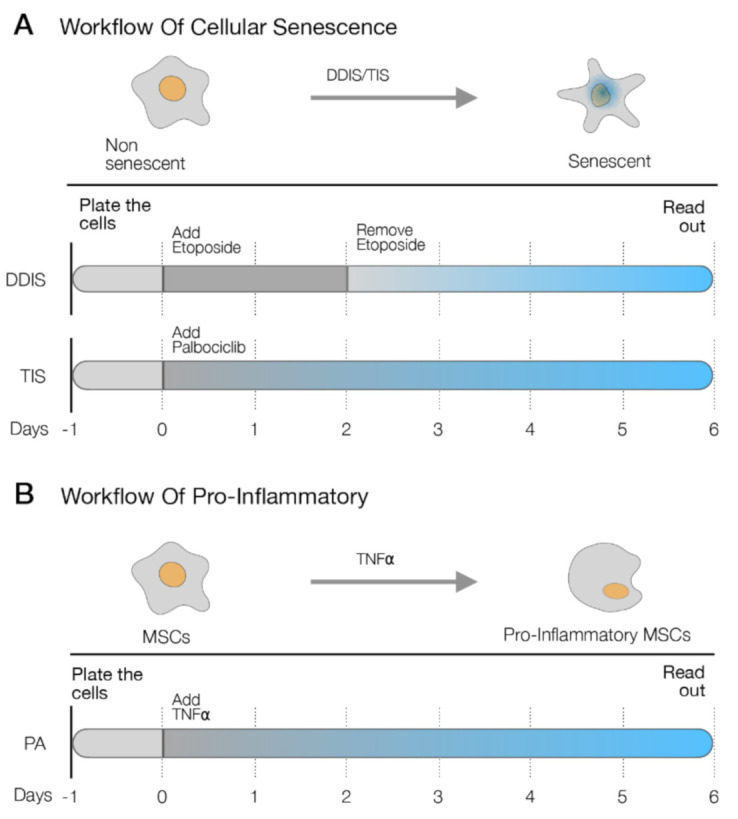
Schematic representation of (**A**) two cellular senescence models in MSCs: DNA damage- induced senescence (DDIS) (up) and therapy-induced senescence (TIS) (bottom) and (**B**) the proinflammatory activation (PA) model in mesenchymal stem cells (MSCs) using tumor necrosis factor α (TNF-α).

**Figure 3:**
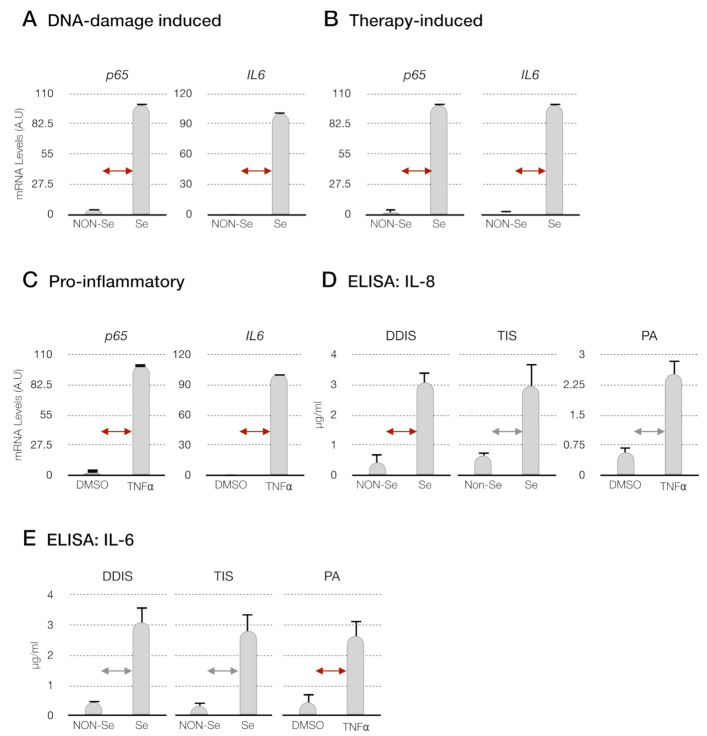
Evaluation of the p65 pathway in cellular senescence and proinflammatory activation; qPCR to determine the mRNA *p65* expression in (**A**) DNA damage-induced senescence (DDIS), (**B**) therapy-induced senescence (TIS) and (**C**) proinflammatory activation (PA) in mesenchymal stem cells (MSCs). Data represent the mean ± SD of three independent experiments. Nonparametric Kolmogorov–Smirnov test was used to calculate the significance, and it was represented as 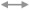 *p* < 0.05 and 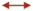 *p* < 0.01 (**D**) levels of interleukin-8 (IL-8) and (**E**) interleukin-6 (IL-6) in the conditioned medium from senescent cells (DDIS and TIS) and proinflammatory-activated MSCs. Data shows the mean ± SD of three independent experiments. Nonparametric Kolmogorov–Smirnov test was used to calculate the significance, and it was represented as 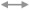 *p* < 0.05 and 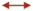 *p* < 0.01. A.U: arbitrary units, NON-Se: proliferative or nonsenescent MSCs, Se: senescent (DDIS or TIS) MSCs, DMSO: MSCs treated with DMSO (dimethyl sulfoxide) and TNF-α: MSCs treated with tumor necrosis factor alpha.

Firstly, we established two cellular senescence models in MSCs: (i) DNA damage-induced senescence (DDIS): MSCs were treated with etoposide for two days. Then, the cells were withdrawn from the etoposide for four days, allowing the cellular senescence phenotype (Figure 2A) and (ii) therapy-induced senescence (TIS): MSCs were treated for six days with palbociclib, a selective inhibitor of cyclin-dependent kinase 4 and 6 (CDK4/6) used to induce cell cycle arrest and the cellular senescence phenotype in cancer cells [24] (Figure 2A). The endogenous expression of *p65* was monitored by quantitative reverse-transcription PCR (qPCR-RT). We obtained a statistically significant increase in the transcription of *p65* in the senescent MSCs (Se(DDIS) and Se(TIS)) in comparison to proliferantive or nonsenescent (Non-Se) MSCS (Figure 3A,B). Besides, we observed a statistically significant high content of proinflammatory-elemented SASP regulated by the NF-kB pathway (interleukin-6 and interleukin-8 (IL-6 and IL-8)), using ELISA in the conditioned medium from Se (DDIS and TIS) (Figure 3D,E).

In parallel, we performed an inflammatory model in MSCs using tumor necrosis factor alpha (TNF-α) involved in the proinflammatory activation (PA) of MSCs after treatment for six days (Figure 2B). We evaluated the expression of *p65* at the RNA level and the proinflammatory components (IL-6 and IL-8) by qPCR-RT and ELISA, respectively. We found that the MSCs treated with TNF-α showed statistically significant high mRNA levels of *p65* (Figure 3C) and high levels of IL-6 and IL-8 in their conditioned medium (Figure 3D,E).

With these data, we confirmed that in the cellular senescence and proinflammatory activation of MSCs exists an activation of the p65 pathway.

### 2.2. Inhibition of p65 Prevents the Cellular Senescence Establishment and Proinflammatory Activation in MSCs

**Figure 4:**
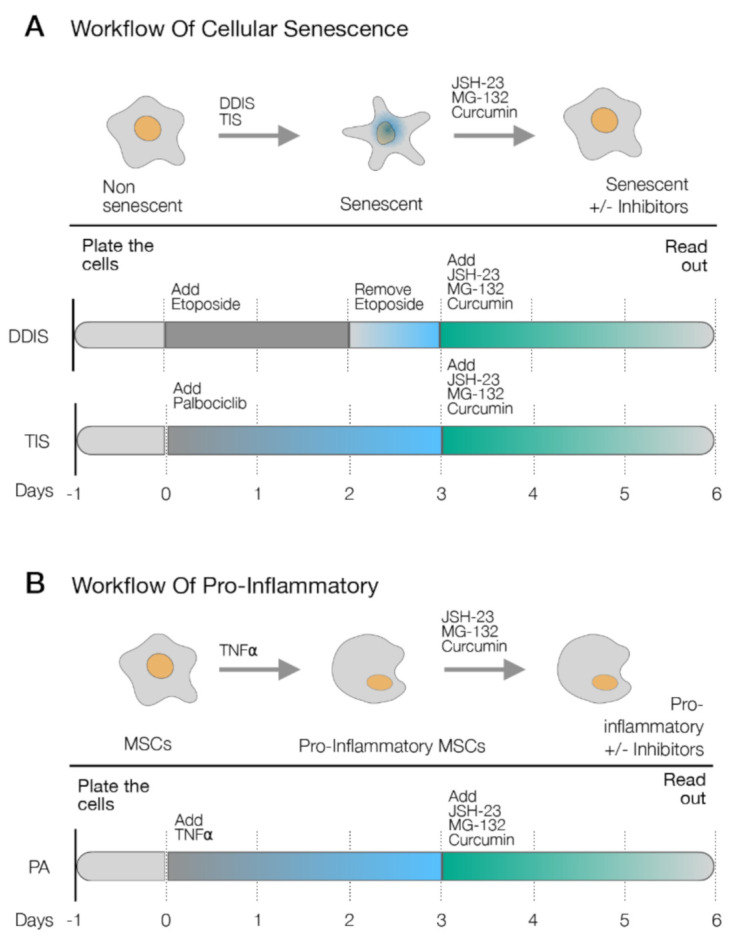
Schematic representation of the experimental settings to determine the role of p65 in (**A**) the cellular senescence models (DNA damage-induced senescence (DDIS) and therapy-induced senescence (TIS)) and (**B**) the proinflammatory activation (PA) in mesenchymal stem cells (MSCs) using the pharmacological inhibitors of the p65 pathway (JSH-23, MG-132 and curcumin). TNF-α: tumor necrosis factor alpha. +/−: with/without the treatment.

To assess the relevance of p65 in the cellular senescence and proinflammatory activation of MSCs, we selected a inhibitor of the p65 pathway (JSH-23, MG-132 or curcumin) [25,26,27] to affect the cellular senescence and proinflammatory activation. In DDIS, after removing the etoposide for one day, cells were treated with the inhibitors of the p65 pathway for three days (Figure 4A). In the DDIS, TIS and PA models, cells were treated with the inhibitors after three days of treatment with palbociclib and TNF-α, respectively (Figure 4A,B).

**Figure 5:**
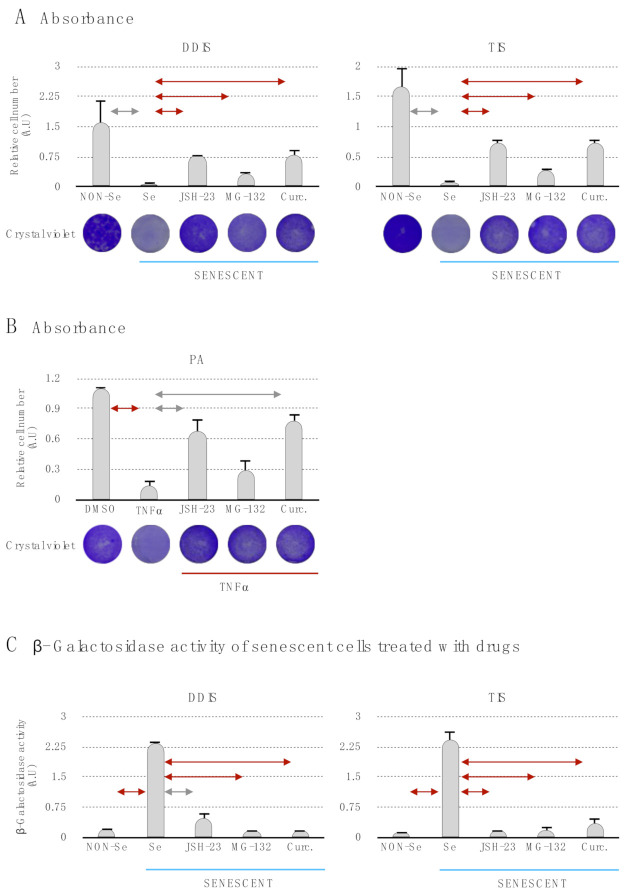
The inhibition of the p65 pathway prevents the induction of cellular senescence and proinflammatory activation. Quantification and representative images of crystal violet to evaluate the proliferation capacity in (**A**) mesenchymal stem cells (MSCs) treated with JSH-23, MG-132 and curcumin during the cellular senescence establishment (DNA damage-induced senescence (DDIS) and therapy-induced senescence (TIS)) and (**B**) MSCs treated with JSH-23, MG-132 and curcumin during the proinflammatory activation (PA). All data represent the mean ± SD of three independent experiments. Nonparametric Kolmogorov–Smirnov test was used to calculate the significance, and it was represented as 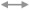 *p* < 0.05 and 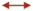 *p* < 0.01. (**C**) Quantification of senescence-associated β-galactosidase activity in MSCs treated with the pharmacological inhibition of the p65 pathway using JSH-23, MG-132 and curcumin during the cellular establishment (DDIS and TIS). All data represent the mean ± SD of three independent experiments. Nonparametric Kolmogorov–Smirnov test was used to calculate the significance, and it was represented as 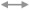 *p* < 0.05 and 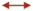 *p* < 0.01. NON-Se: Proliferative or nonsenescent MSCs, Se: Senescent MSCs; TNF-α: MSCs treated with tumor necrosis factor alpha, JSH-23: Senescent (DDIS and TIS) or proinflammatory-activated (TNF-α) MSCs treated with JSH-23, MG-132: Senescent (DDIS and TIS) or proinflammatory activated (TNF-α) MSCs treated with MG-132, Curc.: Senescent (DDIS and TIS) or proinflammatory activated (TNF-α) MSCs treated with curcumin and A.U: arbitrary units.

Then, we evaluated in the DDIS, TIS and PA the proliferation capacity of the cells using crystal violet staining and its quantification (Figure 5A,B). The Se (DDIS and TIS) and TNF-α showed a statistiscally significant decrease of the proliferation capacity in comparison to Non-Se (DDIS and TIS) and DMSO, respectively (Figure 5A,B). Besides, we found that treatment with the inhibitors of the p65 pathway (JSH-23, MG-132 and curcumin) prevents statistically significant cell cycle arrest in DDIS, TIS and PA (Figure 5A,B). Additionally, we evaluated the lysosomal activity by measuring β-gal in the senescence process in MSCs. The levels of β-gal increased statistically significantly in Se (DDIS and TIS) and decreased when the cells were treated with JSH-23, MG-132 or curcumin (Figure 5C). Altogether, these data suggest that p65 is an important pathway in the establishment of cellular senescence and proinflammatory activation on MSCs.

### 2.3. p65 Pathway Modulate the Production of sEV in Senescent and Pro-Inflammatory-Activated MSCs

**Figure 6:**
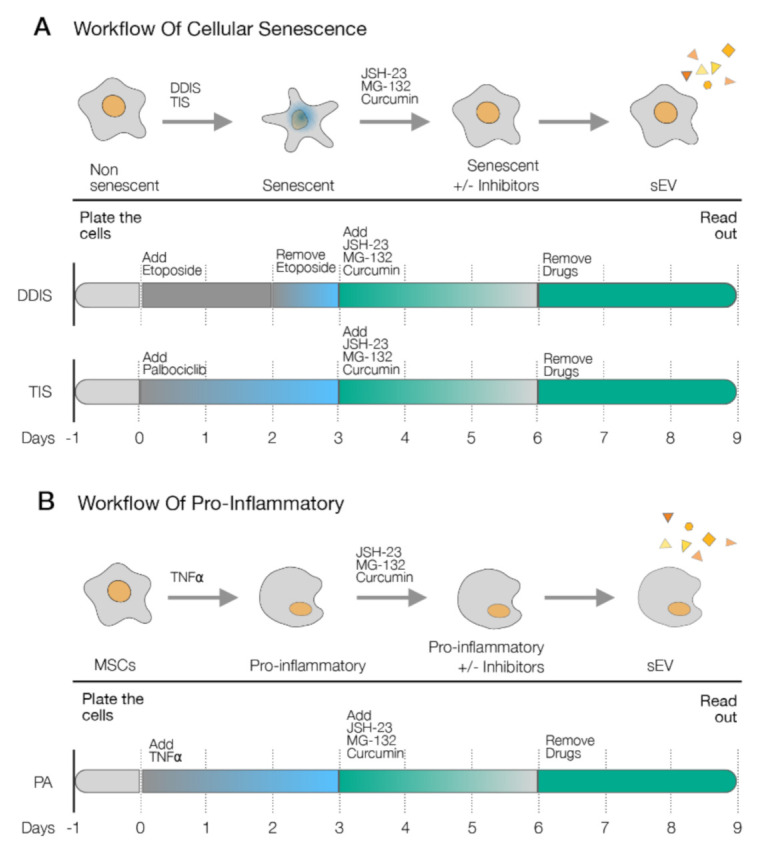
Schematic representation of the experimental settings to determine the role of the p65 pathway in the release of small extracellular vesicles (sEV). sEV were isolated from (**A**) cellular senescence models (DNA damage-induced senescence (DDIS) and therapy-induced senescence (TIS)) and (**B**) proinflammatory activation (PA) in mesenchymal stem cells (MSCs) after the treatment with pharmacological inhibitors (JSH-23, MG-132 and curcumin) during the induction.

In order to determine if the p65 pathway is involved in the release of EV in inflamm-aging, we treated the cells with the drugs to induce cellular senescence (DDIS and TIS) and proinflammatory activation, as previously described, followed by treatment with the inhibitors of the p65 pathway for 72 h. The cells were then washed with PBS to remove the inhibitors, and the sEV were collected three days later (Figure 6A,B).

**Figure 7:**
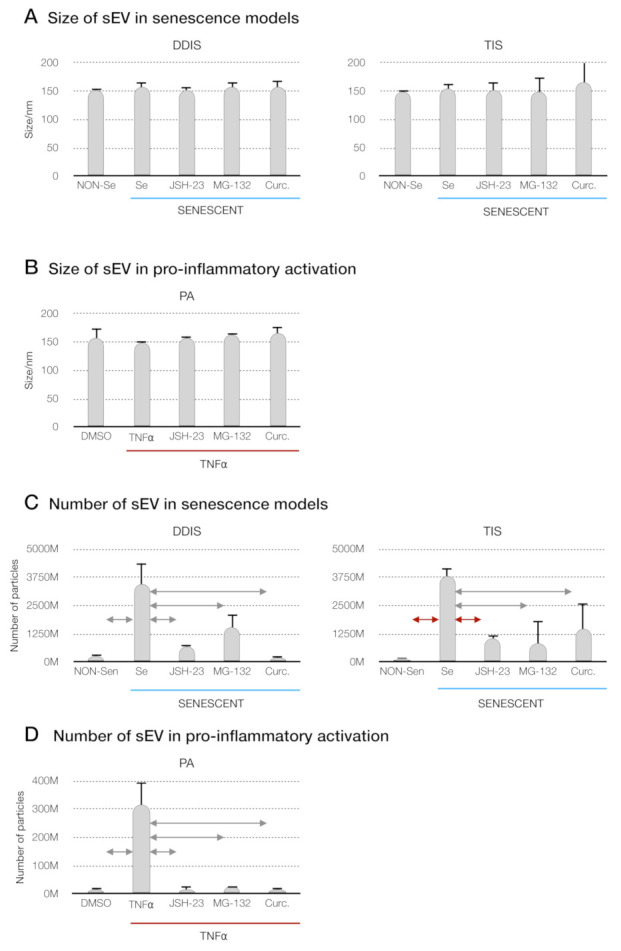
Inhibition of the p65 pathway in cellular senescence and proinflammatory activation in MSCs prevents the release of small extracellular vesicles (sEV). (**A**) Size distribution of sEV from MSCs treated with JSH-23, MG-132 and curcumin during the induction of cellular senescence models (DDIS and TIS) and (**B**) proinflammatory activation (PA) using a nanoparticle tracking analysis (NTA). Data shown the mean ± SD of three independent experiments. Nonparametric Kolmogorov–Smirnov test was used to calculate the significance, and it was represented as 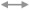 *p* < 0.05 and 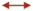 *p* < 0.01. Quantification of the number of particles in (**C**) MSCs treated with JSH-23, MG-132 and curcumin during the cellular senescence induction (DDIS and TIS) and (**D**) PA using an NTA. The graphs shown the mean ± SD of three independent experiments. Nonparametric Kolmogorov–Smirnov test was used to calculate the significance, and it was represented as 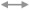 *p* < 0.05 and 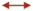 *p* < 0.01. NON-Se: proliferative or nonsenescent MSCs, Se: senescent MSCs, TNF-α: MSCs treated with tumor necrosis factor alpha, JSH-23: senescent (DDIS and TIS) or proinflammatory-activated (TNF-α) MSCs treated with JSH-23, MG-132: senescent (DDIS and TIS) or proinflammatory-activated (TNF-α) MSCs treated with MG-132, Curc.: senescent (DDIS and TIS) or proinflammatory-activated (TNF-α) MSCs treated with curcumin, nm: nanometer and M:1000.

The size of the EV diameter and number of EVs were measured using a nanoparticle tracking analysis (NTA). We observed that the size of the EV diameter was around 150 nm, as in the exosome-like particles of sEV, and the diameter size of the EV was not statistically significant from the Se (DDIS and TIS) and TNF-α-treated MSCs, compared to those treated with JSH-23, MG-132 and curcumin in the different models (Figure 7A,B). With respect to EV number, we found that Se- (DDIS and TIS) and TNF-α-treated MSCs released statistically significantly increased the number of particles compared to Non-Se- and DMSO-treated MSCs, respectively. Besides, treatments with JSH-23, MG132 and curcumin separately prevented a partial release in cellular senescence and proinflammatory activation (Figure 7C,D). These data show that the pharmacological inhibition of the p65 pathway prevents the release of EV from senescent and proinflammatory MSCs.

### 2.4. p65 Is a Common Pathway Involved in the Cellular Senescence and Proinflammatory Activation Paracrine through sEV

**Figure 8:**
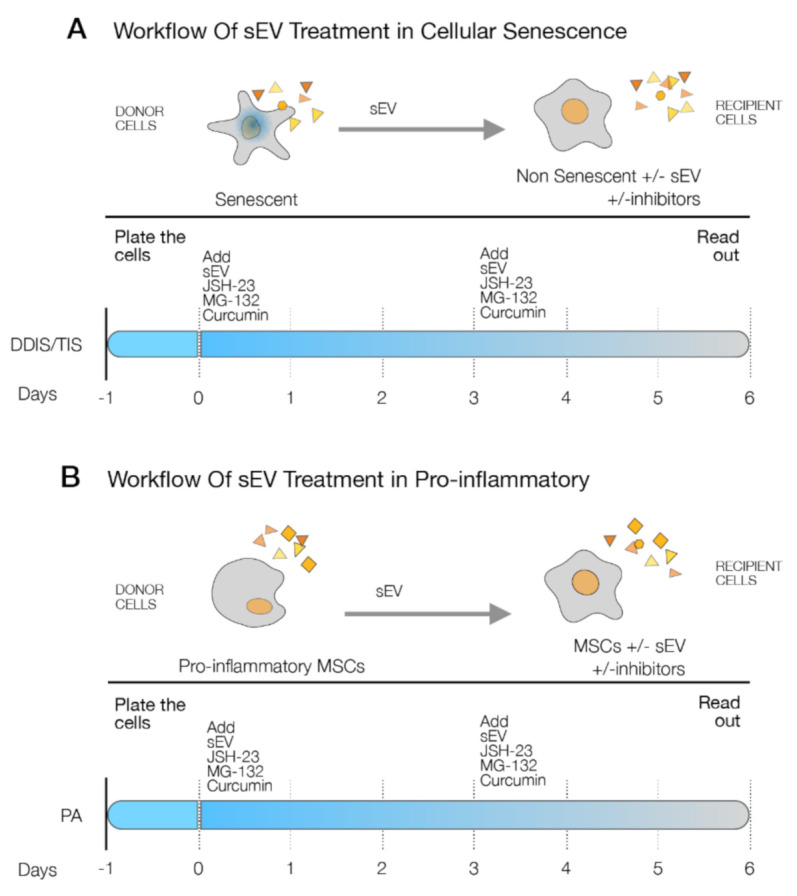
Schematic representation of the details of the experimental settings to the p65 pathway involved in the cellular senescence and proinflammatory paracrine transmission by small extracellular vesicles (sEV). (**A**) Proliferative mesenchymal stem cells (MSCs) (Non-Se) were treated with sEV from Se (DNA damage-induced senescence (DDIS) and therapy-induced senescence (TIS)) and the small inhibitors (JSH-23, MG-132 and curcumin) for six days. (**B**) MSCs were treated with sEV from proinflammatory-activated MSCs (tumor necrosis factor alpha (TNF-α)) and JSH-23, MG-132 and curcumin for six days.

To further determine the implication of the p65 pathway in the paracrine transmission of inflamm-aging, we isolated EV from senescent and proinflammatory MSCs. The recipient proliferative MSCs (Non-Se or MSCs) were treated with sEV from Se (DDIS and TIS) and TNF-α and simultaneously with the pharmacological inhibitors of the p65 pathway (JSH-23, MG-132 and curcumin) for six days (Figure 8A,B).

**Figure 9:**
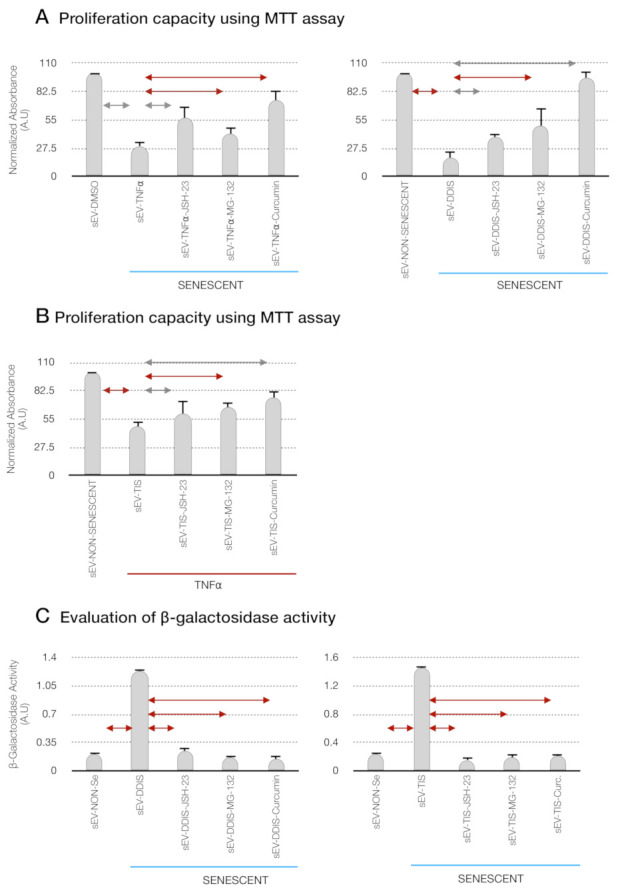
Pharmacological inhibition of the p65 pathway prevents the cellular senescence and proinflammatory paracrine transmission by small extracellular vesicles (sEV) in mesenchymal stem cells (MSCs). Quantification of the proliferation capacity using the MTT (bromide 3-(4,5-dimetiltiazol-2-il)-2,5-diphenyltetrazolium) assay in (**A**) recipient proliferative MSCs (Non-Se) treated with sEV from Non-Se, Senescent MSCs (DDIS and TIS) and JSH-23 (sEV-DDIS-JSH-23 and sEV-TIS-JSH-23), MG-132 (sEV-DDIS-MG-132 and sEV-TIS-MG-132) and curcumin (sEV-DDIS-Curcumin and sEV-TIS-Curcumin) and (**B**) recipient MSCs treated with sEV from DMSO (without TNF-α treatment); TNF-α (tumor necrosis factor alpha treatment) and inhibitor of the p65 pathway (JSH-23, MG-132 and curcumin) (sEV- TNFα-JSH-23, sEV- TNFα-MG-132 and sEV- TNFα-Curc). (**C**) Quantification of senescence-associated β-galactosidase activity in recipient Non-Se treated with sEV from Non-Se, Senescent MSCs (DDIS, and TIS) and JSH-23 (sEV-DDIS-JSH-23 and sEV-TIS-JSH-23), MG-132 (sEV-DDIS-MG-132 and sEV-TIS-MG-132) and curcumin (sEV-DDIS-Curcumin and sEV-TIS-Curcumin). The graphs show the mean ± SD of three independent experiments. Nonparametric Kolmogorov–Smirnov test was used to calculate the significance, and it was represented as 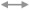 *p* < 0.05 and 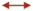 *p* < 0.01. A.U: arbitrary units and MTT (bromide 3-(4,5-dimetiltiazol-2-il)-2,5-diphenyltetrazolium).

The sEV from Se (DDIS and TIS) and TNF-α induced a statistically significant cell cycle arrest measured by the bromide 3-(4,5-dimetiltiazol-2-il)-2,5-diphenyltetrazolium (MTT) assay in comparison with the recipient cells treated with sEV from Non-Se- and DMSO-treated MSCs (Figure 9A,B). The recipient MSCs treated with JSH-23, MG-132 and curcumin prevented the loss of proliferation by sEV from DDIS, TIS and TNF-α (Figure 9A,B). Besides, the pharmacological inhibitor of p65 statistically reduced the significantly high levels of senescence-associated β-gal activity in the paracrine senescence transmission by sEV, as shown in Figure 9C. Altogether, these data suggest that the p65 pathway is involved in the transmission of senescence and proinflammatory activation paracrine through sEV.

## 3. Discussion

Inflamm-aging is a process characterized by the persistent low-grade systemic proinflammatory status producing tissue damage in aging [6]. The senescent and proinflammatory activation of MSCs delivered proinflammatory components, such as IL-6 and IL-8, that positively stimulate inflamm-aging in the body [5]. The process affects the proliferation and self-renewal of MSCs to be used as a therapy in age-related diseases [3,5]. Due to that, we proposed to study the cellular senescence and proinflammatory activation [5] in human MSCs using DDIS, TIS and PA to mimic the physiological status at the in vitro level.

The canonical NF-kB (p65) pathway is activated in senescent cells and functions as a key SASP regulator through p53 and the production of proinflammatory cytokines and chemokines in several types of cells [20,28]. This pathway is involved in the proinflammatory activation of TNF-α in MSCs [21,29]. For this reason, we selected pharmacological inhibitors of the p65 pathway: (i) JSH-23, a well-known selective inhibitor of nuclear translocation of p65 [25], (ii) MG-132, which blocks activation of p65 by preventing degradation of IkB [27] and (iii) curcumin, a naturally derived compound reported to be an effective nuclear p65 inhibitor [26,30]. Firstly, we validated that the induction of cellular senescence (DDIS and TIS) and proinflammatory activation in human MSCs are regulated by the p65 pathway, based on cell proliferation and β-*g*al activity. Our results confirmed Pandey’s results, which reported that expression of the p65 pathway components is increased in aged MSCs from adipose tissue and bone marrow [31] and inflammation-induced TNF-α in MSC-derived bone marrow [29] (Figure 3).

The SASP is the main source of senescent cell communication and microenvironment in the development of age-related diseases. During the last decade, many studies have focused on the therapeutic potential of pharmacological inhibitors of SASP (called senomorphic drugs), like the p65 pathway [32], to disrupt paracrine transmission cellular senescence. Our results coincide with results from Chien et al. in human fibroblasts [33] (Figure 5 and Figure 7). The use of sEV-based therapy from MSCs has a high potential for treating age-related diseases [9], because the sEV cargo is from various cell compartments that mediate intracellular communication via shuttling bioactive signaling molecules or by the direct activation of signaling pathways to recipient cells [11]. For example, the EV from MSCs have immunomodulation effects in immune cells in the lungs in models of asthma, a chronic inflammatory lung disease [34], and bovine milk sEV can ameliorate chronic inflammatory disorders (arthritis and allergy) [35,36].

**Figure 10:**
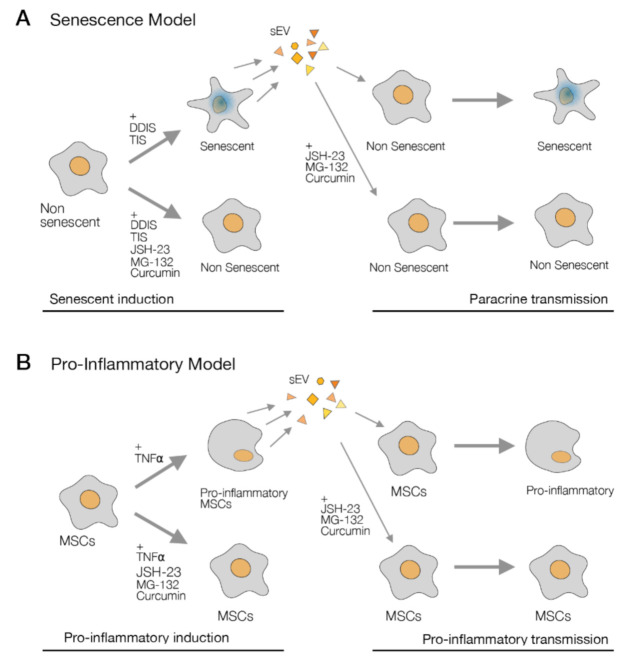
Graphical abstract the role of p65 in inflamm-aging in mesenchymal stem cells (MSCs). **(A**) The use of JSH-23, MG-132 and curcumin blocks the senescence induction (left), and the inhibition of the p65 pathway prevents the paracrine senescence transmission by sEV in human MSCs (right). (**B**) The p65 pathway is involved in the proinflammatory activation in MSCs (left) and the proinflammatory transmission paracrine by small extracellular vesicles (sEV) (right). DDIS: DNA damage-induced senescence, TIS: therapy-induced senescence and TNF-α: tumor necrosis factor alpha.

However, the mechanism that can affect the therapeutic potential is not clear. In the last years, it has been identified that sEV can mediate paracrine senescence in different contexts using human fibroblasts [10] and bone marrow MSCs [12]. We confirmed that the transmission of paracrine senescence (DDIS and TIS) by sEV in human MSCs can occur, and we discovered that sEV from proinflammatory-activated MSCs can induce cell cycle arrest in normal MSCs (Figure 9 and Figure 10). Both processes are regulated by the p65 pathway in MSCs, which we showed in the functionality experiment with the inhibitors.

The pharmacological inhibitors of the p65 pathway (JSH-23, MG-132 and curcumin) have high potential to use comparable senomorphic drugs to show a decrease in the release of sEV in senescent- and proinflammatory-activated MSCs (Figure 7). With our results, these inhibitors could prevent cellular senescence and inflammation in MSCs characterized by inflamm-aging. The inhibitors of the p65 pathway could be used as senomorphic drugs to inhibit the inflamm-aging effect in MSCs (Figure 10) and maintain the stem cell properties of MSCs in in vitro cultures to use as therapy in age-related diseases like atherosclerosis [37]. The study of cargo sEV (protein, lipids and RNAs) involved in a therapy capacity regulated by the inhibition of the p65 pathway could be a promising point to advance the knowledge of sEV-based therapy, because the p65 pathway is related with age-related diseases like osteoarthritis [38].

A limitation of the current study is the performance of only in vitro studies and the short time studied. Longer studies will need to be performed and validated in in vivo models. However, this study is a proof-of-concept to use the pharmacological inhibitors of p65 (JSH-23, MG-132 and curcumin) to maintain the properties of MSCs and their sEV throughout their aging. Besides, they can decrease the accumulation of senescent- and proinflammatory-activated MSCs in the body to delay the development of age-related diseases.

## 4. Materials and Methods

### 4.1. Cell Culture

Human umbilical cords (UC) were obtained from caesarean sections performed on healthy women at the Maternity Facility at Complejo Hospitalario Universitario A Coruña (CHUAC). All tissues were obtained with fully informed consent and ethical approval by the supervisor of the Ethical Committee (CEIC: 2019/026) of Galicia. All the women were between 26–35 years of age. Mesenchymal stem cells (MSCs) were isolated from UC using the protocol developed by Arufe’s group [1]. Briefly, the tissue was washed with phosphate-buffered saline and cut into small pieces (explants). These explants were then incubated for three five-minute periods in an enzyme mixture containing 1.2-U/mL dispase and 112-U/mL type I collagenase (all from Sigma-Aldrich, Madrid, Spain) and cultured in Dulbecco′s Modified Eagle’s Medium with 10% (*v/v*) fetal bovine serum, 1% (*v/v*) penicillin and 1% (*v/v*) streptomycin (all from Sigma-Aldrich, Madrid, Spain) and growth adhered to the plastic plate. After three days, the explants were removed from the plate, leaving the attached UC-MSCs, which were then cultured in a monolayer in the same medium. When the cells were in passage 4 and 90% confluent, they were removed from the plate using 2% (*v/v*) trypsin (Sigma-Aldrich, Madrid, Spain) in phosphate-buffered saline to seed in the plates to perform the experiments. T/C-28a2 (TC28a2) immortalized healthy chondrocytes from human costal cartilage from a 15-year-old female (SCC042) (Sigma-Aldrich, Madrid, Spain) were cultured in Dulbecco′s Modified Eagle’s Medium with 10% (*v/v*) fetal bovine serum, 1% (*v/v*) penicillin and 1% (*v/v*) streptomycin (all from Sigma-Aldrich, Madrid, Spain).

### 4.2. Cellular Senescence Induction in UC-MSCs

Cellular senescence was induced in proliferative UC-MSCs using two models: (1) DDIS: the UC-MSCs were cultured with DMEM supplemented with 10% (*v/v*) FBS, 1% (*v/v*) penicillin, 1% (*v/v*) streptomycin (all from Sigma-Aldrich, Madrid, Spain) and 1-μM Etoposide (MedChemexpress, New Jersey, USA) (Table A1) for two days. The cells were then washed with PBS and cultured with DMEM supplemented with 10% (*v/v*) FBS, 1% (*v/v*) penicillin and 1% (*v/v*) streptomycin (all from Sigma-Aldrich, Madrid, Spain) for four days (Figure 2A). (2) TIS: the UC-MSCs were cultured with DMEM supplemented with 10% (*v/v*) FBS, 1% (*v/v*) penicillin, 1% (v/v) streptomycin (all from Sigma-Aldrich, Madrid, Spain) and 1-μM Palbociclib (CDK4/6 inhibitor) (MedChemexpress, New Jersey NJ, USA) (Table A1) for three days. After that, the cells were washed with PBS and cultured with DMEM supplemented with 10% (*v/v*) FBS, 1% (*v/v*) penicillin and 1% (*v/v*) streptomycin (all from Sigma-Aldrich, Madrid, Spain) for three days. The cells were then washed with PBS and cultured with DMEM supplemented with 10% (*v/v*) FBS, 1% (*v/v*) penicillin and 1% (*v/v*) streptomycin (all from Sigma-Aldrich, Madrid, Spain) for three days (Figure 2A).

### 4.3. Pro-Inflammatory Activation in UC-MSCs

UC-MSCs were incubated with 5-ng/mL TNF-α recombinant (Immunotools, Gladiolenweg, Germany) in DMEM supplemented with 10% (*v/v*) FBS, 1% (*v/v*) penicillin and 1% (*v/v*) streptomycin for three days. After that, the cells were washed with PBS and were cultured with DMEM supplemented with 10% (*v/v*) FBS, 1% (*v/v*) penicillin and 1% (*v/v*) streptomycin (all from Sigma-Aldrich, Madrid, Spain) for three days (Figure 2B).

### 4.4. Treatment with Drugs

MSCs were seeded at a 50% confluence. The next day, the induction of senescence and inflammatory activation was started, as previously described. Three days later, the cells were treated with drugs at suitable concentrations (Table A1) for six days.

### 4.5. Treatment with sEV

The proliferative UC-MSCs were plated. One day later, the cells were treated with sEV from donor cells for six days with the same number of sEV in DMEM 10% (*v/v*) FBS-depleted sEV and 1% (*v/v*) penicillin and 1% (*v/v*) streptomycin (all from Sigma-Aldrich, Madrid, Spain).

### 4.6. Cell Proliferation Assay

The number of cells was evaluated by the colorimetric MTT assay (Cell Proliferation Kit I, Roche, Basel, Switzerland) according to manufacturer’s instructions. The absorbance was measured with a NanoQuant microplate reader (Tecan Trading AG, Männedorf, Switzerland) at 540 nm.

### 4.7. Crystal Violet Staining

The plates were fixed using 4% (*v/v*) paraformaldehyde for 15 min at room temperature and washed three times with PBS. Then, the plates were stained with 0.5% (*v/v*) crystal violet solution and scanned. The quantification of crystal violet was performed by solubilizing crystal violet staining (Sigma-Aldrich, Madrid, Spain) with 30% (*v/v*) acetic acid and measuring the absorbance at 570 nm using a NanoQuant microplate reader (Tecan Trading AG, Switzerland).

### 4.8. β-GALACTOSIDASE Assay

β-gal activity was measured using the Thermo Scientific Mammalian β-Galactosidase Assay Kit (Thermo Fisher, Foster City, CA, USA), following the manufacturer’s instructions, at 405 nm using a NanoQuant microplate reader (Tecan Trading AG, Switzerland).

### 4.9. Isolation Extracellular Vesicles

The cells were cultured with DMEM supplemented with 10% (*v/v*) sEV-depleted FBS and 1% (*v/v*) penicillin and 1% (*v/v*) streptomycin (all from Life Technologies, Carlsbad, CA, USA). Cells were cultured to 80% confluence, and the supernatants were collected after 72 h. Supernatants were centrifuged at 2000× *g* for 10 min at 4 °C and filtered using a sterile 0.22-μm filter (GE Healthcare Life Sciences, Little Chalfont, UK) to eliminate debris, and they were transferred into new ultracentrifugation tubes (Beckman Coulter, Mississauga, ON, Canada) and centrifuged at 100,000× *g* for 2 h at 4 °C in an Optimal-90K ultracentrifuge with a 60 Ti rotor (Beckman Coulter, Mississauga, ON, Canada). The last supernatants containing exosome-depleted FBS were removed, and the pellets were resuspended in 200-μL PBS (MP Biomedicals, Illkrich-Graffenstaden, France).

### 4.10. RNA Isolation, Synthesis cDNA and qPCR-RT

Total RNA was extracted using TRIzol Reagent (Thermo Fisher, Foster City CA, USA) according to the manufacturer’s instructions. cDNA synthesis was performed using a High-Capacity cDNA Reverse Transcriptase kit (Thermo Fisher, Foster City, CA, USA). qPCR reactions were performed using SYBR Green PCR Master Mix (Applied Biosystems, Foster City, CA, USA) and the primers in the Table A3 using LightCycler 480 (Roche, Basel, Switzerland).

### 4.11. Flow Cytometry

UC-MSCs were washed twice in PBS and pre-blocked with 2% (*v/v*) rat serum in PBS. The cells were incubated with the primary antibody at suitable concentrations (Table A2) for 1 h at room temperature. After incubation, the cells were washed twice with PBS. The FACS data was generated by BD FACSDiva software (BD Science, San Jose, CA, USA). Negative control staining was performed using the isotype (Table A2).

### 4.12. ELISA

The supernatant from UC-MSCs were diluted 1:2 prior to analysis. Human IL-6 and IL-8 immunoassays (h-IL6-EIA-1 and h-IL8-EIA-5, respectively, Mab Tag GmbH) were performed following the manufacturer’s instructions. The absorbance from the samples was measured at 540 nm using a NanoQuant microplate reader (Tecan Trading AG, Switzerland).

### 4.13. Nanoparticle Tracking Analysis

The Brownian motion of the particles in a NanoSight LM12 using Nanoparticle Tracking Analysis 2.3 software (NanoSight Ltd., Amesbury, UK) was used to calculate the EV size distribution after the ultracentrifugation.

### 4.14. Statistics

Data are expressed as the mean ± SD, and the statistical analysis was performed, considering the groups compared were unpaired and using the nonparametric Kolmogorov–Smirnov test with GraphPad Prism Version 9.0.1. 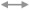 *p* < 0.05 and 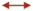 *p* < 0.01 were considered statistically significant.

## 5. Conclusions

In this study, we discovered that the modulation of the NF-kB pathway by the inhibition of p65 using small inhibitors (JSH-23, MG-132 and curcumin) can prevent cellular senescence and proinflammatory activation in MSCs and modulate the emerging SASP. Additionally, a small inhibitor treatment can prevent paracrine and proinflammatory transmission by sEV.

## Appendix A

**Table A1:** List of drugs.

Drug	Concentration	Source	Cat. No
Etoposide	1 µM	MedChemExpress	HY-13629
Palbociclib (CDK4/6i)	1 µM	MedChemExpress	HY-50767
TNF-A α	10 ng/mL	ImmunoTools	11343013
JSH-23	10 µM	MedChemExpress	HY-13982
MG-132	10 µM	MedChemExpress	HY-13259
Curcumin	10 µM	Sigma-Aldrich	458-37-7

**Table A2:** List of antibodies.

Antibody	Dilution	Source
CD90-FITC	1:100	BD Pharmigen
CD45-FITC	1:100	BD Pharmigen
CD73-PE	1:100	BD Pharmigen
CD105-FITC	1:100	BD Pharmigen
CD34-PE	1:100	BD Pharmigen
IgG1-PE	1:100	St. Cruz Biotechnology’s
IgG1-FITC	1:100	St. Cruz Biotechnology’s

**Table A3:** List of primers.

Target	Species	Forward Primer	Reverse Primer
*p65*	Human	ttcccgatctgagtccaggt	gcttgtctcgggtttctgga
*Nanog*	Human	atgcctcacacggagactgt	aagtgggttgtttgcctttg
*Sox2*	Human	ctccgggacatgatcagc	ggtagtgctgggacatgtgaa
*Rex1*	Human	cgcaatcgcttgtcctcagagt	gctctcaacgaacgctttccca
*Oct4*	Human	ctcctggagggccaggaatc	atatacacaggccgatgtgg
*β* *-actin*	Human	agagctacgagctgcctgac	ggatgccacaggactcca
